# Biopolymer Composites with Ti/Au Nanostructures and Their Antibacterial Properties

**DOI:** 10.3390/pharmaceutics13060826

**Published:** 2021-06-02

**Authors:** Petr Slepička, Dominik Fajstavr, Markéta Krejčová, Silvie Rimpelová, Nikola Slepičková Kasálková, Zdeňka Kolská, Václav Švorčík

**Affiliations:** 1 Department of Solid State Engineering, University of Chemistry and Technology Prague, 166 28 Prague, Czech Republic; dominik.fajstavr@vscht.cz (D.F.); krejcovamiki@seznam.cz (M.K.); nikola.kasalkova@vscht.cz (N.S.K.); vaclav.svorcik@vscht.cz (V.Š.); 2 Department of Biochemistry and Microbiology, University of Chemistry and Technology Prague, 166 28 Prague, Czech Republic; 3 Faculty of Science, J. E. Purkyně University in Ústí nad Labem, 400 96 Ústí nad Labem, Czech Republic; zdenka.kolska@ujep.cz

**Keywords:** biopolymer, wrinkled patterns, nanostructured surface, antimicrobial activity, surface morphology, antibacterial properties, composites, titanium layer, gold layer, thermal modification

## Abstract

In this study, we have aimed at the preparation and characterization of poly-l-lactic acid (PLLA) composites with antibacterial properties. Thin bilayers of titanium and gold of various thickness ratios were deposited on PLLA by a cathode sputtering method; selected samples were subsequently thermally treated. The surface morphology of the prepared composites was studied by atomic force, scanning electron, and laser confocal microscopy. The chemical properties of the composites were determined by X-ray photoelectron and energy-dispersive X-ray spectroscopy in combination with contact angle and zeta potential analyses. The antibacterial properties of selected samples were examined against a Gram-negative bacterial strain of *E. coli*. We have found that a certain combination of Au and Ti nanolayers in combination with heat treatment leads to the formation of a unique wrinkled pattern. Moreover, we have developed a simple technique by which a large-scale sample modification can be easily produced. The dimensions of wrinkles can be tailored by the sequence and thickness of the deposited metals. A selected combination of gold, titanium, and heat treatment led to the formation of a nanowrinkled pattern with excellent antibacterial properties.

## 1. Introduction

Polymer metallization may lead to an internal tension, which is created between a film and a substrate [1]. Additional stress may be caused by external conditions, such as temperature changes, ambient humidity, external loads, or oxidation. Exceeding the critical tension can then lead to surface deformation and wrinkle formation [2]. The value of critical stress depends on the ratio of Young’s modulus of a film and substrate. This ratio is very low for a polymer-metal system. The critical stress is too low and, therefore, it is very easy to produce a wrinkle-patterned surface structure. [3]. Since tension in a homogeneous film is uniform, the resulting wrinkles are randomly oriented. Only at the edges of the substrate, there is anisotropic stress, when the parallel component is significantly larger than the perpendicular one, and thus, the resulting wrinkles are directed perpendicularly to the edges of the film. With increasing distance from the edges, the anisotropy disappears [2]. A similar anisotropy of stress occurs in bends that may arise in the initial phase of sputtering when metal atoms directly bombard the substrate surface. This results in high compression stresses resulting in cross-linked bends, for example, for polydimethylsiloxane (PDMS). With the development of wrinkles, the bends gradually disintegrate and eventually completely disappear [4]. Sputtered thin films on PDMS revealed that for different metals, a similar behavior occurs, and a wrinkled structure is formed during sputtering [3,4]. A comparison of structures formed after sputtering of different metals has shown that the wavelength of wrinkle pattern depends on both the substrate used and the properties of the deposited metal and that it increases with the growing thickness of a metallic film.

Heat treatment of selected biaxially oriented biopolymers, such PLLA, when heated, may induce a surface wrinkle pattern similar to PDMS [5]. The possibility of such pattern formation was previously confirmed for selected noble metals [6,7]. The wrinkle pattern wavelength in a combination with the surface roughness may affect the properties of the modified polymer, such as its wettability [5]. The appearance of the wrinkles may be induced by heating of the polymer containing a thin metallic layer above its transformation temperature, such as the glass transition temperature (Tg) or the melting temperature (Tm). By reaching the Tg, the glass-to-viscoelastic or viscous state of the polymer changes. By subsequent cooling of the material, a significant compression tension is created in the film due to the large difference in the thermal expansion coefficient of the polymer and the metallic layer which leads to wrinkle formation [6]. It was shown that the thermal stress at the polymer Tg, at certain thicknesses of the metal, results in wrinkle formation with a preferred orientation [7]. The wrinkled structure resulting from thermal stress can be affected by the plasma treatment of the polymer before the metal deposition itself. In both cases, the wrinkled structure depends on the deposited metal thickness. The wrinkle formation mechanism for plasma-modified samples may differ from the unmodified samples [8], with the major difference based on heat instability due to the application of excimer laser treatment [9,10,11,12,13,14]. Mass redistribution is common for both phenomena. For plasma-treated samples, before metallization, two different wrinkles can be formed on the polymer surface. A primary pattern induced mainly due to surface instability from atomic bombardment during sputtering has a wavelength-independent of sputtering time. A secondary structure induced by temperature changes of the system exhibits a wavelength increase with the growing thickness of the metallic layer [4].

In addition to metal deposition and heat treatment, other techniques for creating wrinkled structures exist: plasma treatment, ultraviolet or ozone radiation, electrical induction, mechanical stretching, and solvent swelling [15,16]. A pattern with a desired wavelength, wave amplitude, and other properties adapted to the particular system can be formed by setting external conditions important for applications such as flexible electronics, optics, microfluidic devices, surface engineering, or measurement techniques [17,18]. Thin films have found also an important application in the production of solar cells [19,20]. The advantage of using thin films is in the minimum amount of material used while achieving high efficiency of solar radiation conversion. TiO_2_ thin films on ceramic substrates are used as gas sensors for the detection of methane or carbon monoxide in industrial buildings and homes. The thin films of noble metal nanostructures are also found to be used as high sensitive polluting gas (VOCs) sensors and have a long shelf life as shown in [21,22,23]. The efficiency of the sensors can be increased by doping a thin layer of magnesium oxide [24]. In optics, there is an effort to create anti-reflective coatings that are effective not only against visible light but also against ultraviolet radiation. UV radiation transmits higher energy than visible light and can, therefore, cause health problems after long-term exposure. In addition to being used in glasses or windows, antireflective coatings also find application in solar cells, photonic devices, or chemical agents [25,26]. Depending on the material selected, the coatings may additionally have, for example, passivating or superhydrophilic properties, which may improve the function of the coated material. Protective metallic, inorganic, organic, or hybrid coatings provide barrier protection to materials exposed to severe conditions, such as liquids or chemicals [27].

Thin films are widely used for decorative purposes. Promising candidates in this area are nanocomposites utilizing surface plasmon resonance of noble metal nanoparticles dispersed in dielectric matrices [28,29,30,31,32]; additionally, carbon-polymer composites have been recently utilized in tissue engineering applications [33,34,35]. Three-layer films of newly created metallic dielectrics consisting of two metallic layers (Cr, Al) separated by a dielectric (SiO_2_) layer able to change color were reported to rely on the viewing angle [36]. In recent years, there has been intensive research in the nanomedicine field. Nanocomposite thin films with high biocompatibility and optimal mechanical properties, which may be coated with established implants, are being investigated [37,38,39]. Increased attention has been drawn to amorphous thin-film alloys, which can serve as biocompatible and antimicrobial coatings of stainless steel articles, or improve their mechanical properties [40,41]. In addition to metal-based nanocomposite materials, thin layers of biopolymers are also used in the medical industry. The thin film of the polymer matrix is suitable for targeted drug delivery, which can greatly increase the efficacy of the medicinal product over application in another dosage form. Thus, cellulose, poly(vinyl alcohol), or poly(ethylene oxide) polymers are commonly used [42].

This work focuses on the preparation of thin metal bilayers on a polymer and their analysis and assessment of antibacterial properties. PLLA/Ti/Au and PLLA/Au/Ti systems with different ratios of thicknesses of individual metallic layers were created by a sputtering method on a PLLA substrate, at which the total thickness of metals was 50 nm and 30 nm, respectively. Furthermore, the effect of thermal stress on the surface morphology and properties of the prepared samples was investigated. Heating the PLLA with a thin metallic layer to the glass transition temperature of the polymer led to the formation of a wrinkled structure. To the best of our knowledge, a selective combination of titanium and noble metal nanolayers, which would lead to the formation of a wrinkle-like pattern with outstanding antibacterial properties, has never been studied so far, with titanium dioxide playing an important role in antibacterial nanolayer formation.

## 2. Materials and Methods

### 2.1. Materials and Modification

Poly-(l)-lactic acid (PLLA) in the form of a polymer film was used as a substrate, supplied by Goodfellow (film thickness 50 µm, density 1.25 g∙cm^−3^, glass transition temperature (Tg) = 60 °C, crystallinity between 60 and 70%). To prepare the gold and titanium layers, 99.999% pure metal targets were used.

The metals were applied to the polymer by sputtering by Quorum Q300T ES. Sputtering was performed in an Ar atmosphere (purity of 99.997%). The metal deposition was conducted at a pressure of 0.1 Pa. A current of 20 mA and a sputtering time of 20 to 700 s were set for the gold deposition. A current of 150 mA was set for titanium deposition; the target sample distance was 20 cm with no rotation. Its thickness was monitored using a crystal gauge. Before sputtering, the surface oxide layer was removed from the titanium by “blind” sputtering. Two series of samples were prepared: one with titanium as the primary metal onto which the gold was deposited so that the total thickness was 50 nm; the second series consists of samples with a gold base on which titanium was deposited so that the total thickness was 30 nm. All detailed Ti and Au layer thicknesses are given in the Table 1. Thicknesses were verified by a combination of a scratch test and atomic force microscopy.

Selected samples were thermally stressed in a Binder Owen thermostat oven at 60 °C correspondings to the PLLA glass transition temperature for one hour and then cooled in the air to room temperature.

### 2.2. Analytical Methods

The surface structure was monitored using an Olympus Lext confocal microscope. The surface of heated and unheated samples was monitored using different objectives. The images were processed by OLS software by a PC microscope.

Sample surface morphology was examined by atomic force microscope (AFM) Dimension ICON (Bruker Corp., Billerica, MA, USA); ScanAsyst mode in the air was used for determination. Silicon Tip on Nitride Lever SCANASYST-AIR with a spring constant of 0.4 N∙m^−1^ was used. NanoScope Analysis software was applied for data processing. The mean roughness values (Ra) represent the average of the deviations from the center plane of the sample.

SEM scans were acquired by a scanning electron microscope (FIB-SEM, LYRA3 GMU, Tescan, Brno, Czech Republic). The applied acceleration voltage was 10 kV. The examined samples were covered with a Pt conductive layer of 20 nm thickness by the deposition from the Pt target (purity of 99.9995%, SAFINA, Vestec, Czech Republic). The deposition was performed using the diode sputtering technique (Quorum Q300T equipment, Lewes Road Laughton, UK). Focused ion beam (FIB) cuts were made by a Ga ion beam integrated into the adapted scanning electron microscope. The FIB-SEM images were taken at an angle of 54.8°. The elemental composition was determined by energy-dispersive X-ray spectroscopy (EDS, analyzer X-MaxN, 20 mm^2^ SDD detector, Oxford Instruments, Abingdon, UK). The accelerating voltage for SEM-EDS measurement was 10 kV.

The surface concentration of the oxygen and carbon in the modified substrates was examined by X-ray photoelectron spectroscopy (XPS). The spectra were measured using experience with polymer analysis by XPS, it means using relatively low power of X-ray source (75 W), using monochromatic X-ray radiation (1486.7 eV), and using very low energy of charge compensating electrons (typically 2 eV), all of these to protect the measured polymer surface against changes caused by radiation. The data were processed by the Casa XPS program and the measured spectra were compared with other our results and reference analysis. Peak fitting was based on possibilities of the Casa XPS program; background removal was applied by Shirley curve, which allowed us to respect the asymmetry of the analyzed peaks.

Sample wettability was characterized by goniometry using a SEE System 2020 (v. 2.1. SEE System, Advex Instruments, Brno, The Czech Republic). Drops of water and glycerol with a volume of 8 μL were applied to the samples using an automatic micropipette. Photographs of the drops were taken with the See software; the contact angle was determined by a three-point method. The surface energy of all samples was determined by the Owens–Wendt method. Furthermore, an electrokinetic analysis (zeta potential determination) was performed on a SurPASS Instrument (Anton Paar GmbH, Gratz, Austria) by two methods (streaming current and streaming potential) using two equations (Helmholtz–Smoluchowski, HS, and Fairbrother–Mastins, FM). Samples of 2 × 1 cm^2^ were prepared for the measurement. The samples were studied inside an adjustable gap with an electrolyte of 0.001 mol∙dm^−3^ KCl at a constant pH value of 7.0 and room temperature. Two samples of each type were measured four times with a relative error of up to 5%.

### 2.3. Antibacterial Activity

The antimicrobial properties of the selected samples were determined. Measurements were performed using an environmental Gram-negative bacterial strain of *E. coli* (DBM 3138). The evaluated samples were placed in Falcon tubes with bacterial suspension diluted with physiological solution (0.9% saline) and gently mixed (Shaker Fisher Vortex-Genie 2, Scientific Industries, Bohemia, NY, USA). The samples were left in contact with the bacterial suspension for 4 and 24 h. After the incubation period, the samples were gently mixed again and 25 µL drops were taken from each tube five times (per one plate) and plated on Petri dishes (in triplicates) containing Luria-Bertani (LB) agar. Then, the agar plates with the bacteria were cultivated for 24 h at room temperature, after which photographs of the plates were taken and the number of colony-forming units (CFU) was calculated using ImageJ software (v. 2.1., LOCI, University of Wisconsin, Madison, WI, USA).

## 3. Results

### 3.1. Sample Surface Morphology by LCM Analysis

**Figure 1:**
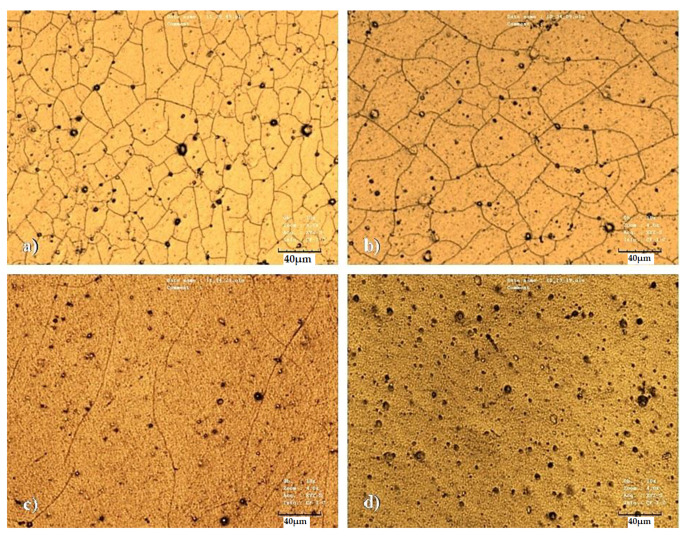
Surface morphology of unheated PLLA samples with sputtered Au and Ti layers. The images were taken by a laser confocal microscope. (**a**) Au and Ti layers of 2 and 28 nm, respectively; (**b**) Au and Ti layers of 5 and 25 nm, respectively; (**c**) Au and Ti layers of 10 and 20 nm, respectively; (**d**) Au and Ti layers of 25 and 5 nm, respectively.

The main idea of this study was a development of a very simple but unique technique of how to prepare a wrinkle-like patterned PLLA substrate with gold and titanium nanolayers enhancing the antibacterial properties of the biopolymer surface. As part of the study of thermally stressed biopolymer composites, we firstly focused on the evaluation of the material surface morphology and changes in biopolymers caused by thermal stress. For this purpose, laser confocal microscopy (LCM) was chosen as the first preliminary method to study changes in the material surface morphology. Figure 1 shows images of the surfaces of unheated samples, in which a primary Au layer was applied to the samples, on which a titanium layer was deposited so that the total metal thickness was set to 30 nm. The images show the formation of a typical structure for titanium nanolayers, i.e., the formation of certain “cracks” in the layer, which occurred during sputtering. The major effect of the titanium layer, which has a thickness of 28 nm in Figure 1a and which gradually fades from 25 and 20 to 5 nm (Figure 1b–d), is evident. It should also be mentioned that the titanium particles have high energy due to the deposition parameters when they hit the substrate, which can also lead to a local increase in the temperature of the sputtering head. Figure 1d shows the most pronounced porosity of the PLLA surface after the application of the metallic layers. We have also studied the surface of unheated titanium-based samples on which gold was deposited to the total thickness of 50 nm (i.e., the metals were deposited in reverse order). The influence of the titanium layer on the material surface morphology was determined to be similar as in Figure 1, with titanium as the bottom layer and gold at the top layer, which led to the formation of a heterogeneous surface structure with microcracks, which are closely related to the thickness of the Au layer, respectively to the different mechanical properties of gold and titanium layer. At higher values of Au thickness, it achieves more homogeneous properties in terms of surface morphology compared to the Ti layer and the surface, therefore, has fewer surface irregularities.

**Figure 2:**
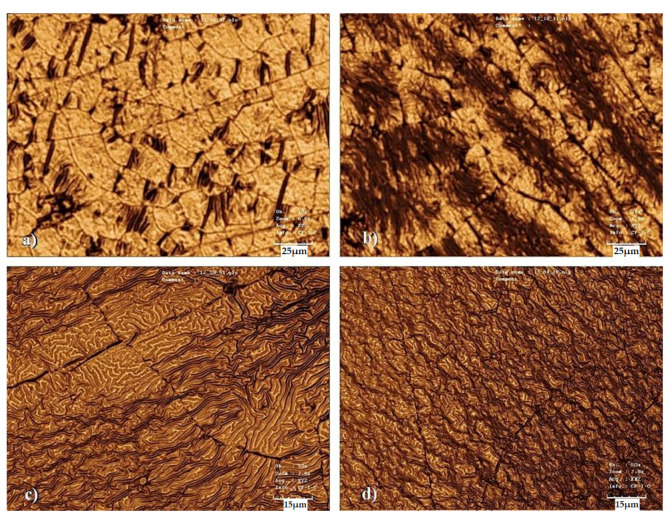
Surface morphology of heated PLLA samples with sputtered Au and Ti layers. The images were taken by a laser confocal microscope. (**a**) Au and Ti layers of 2 and 28 nm, respectively; (**b**) Au and Ti layers of 5 and 25 nm, respectively; (**c**) Au and Ti layers of 10 and 20 nm, respectively; (**d**) Au and Ti layers of 25 and 5 nm, respectively.

**Figure 3:**
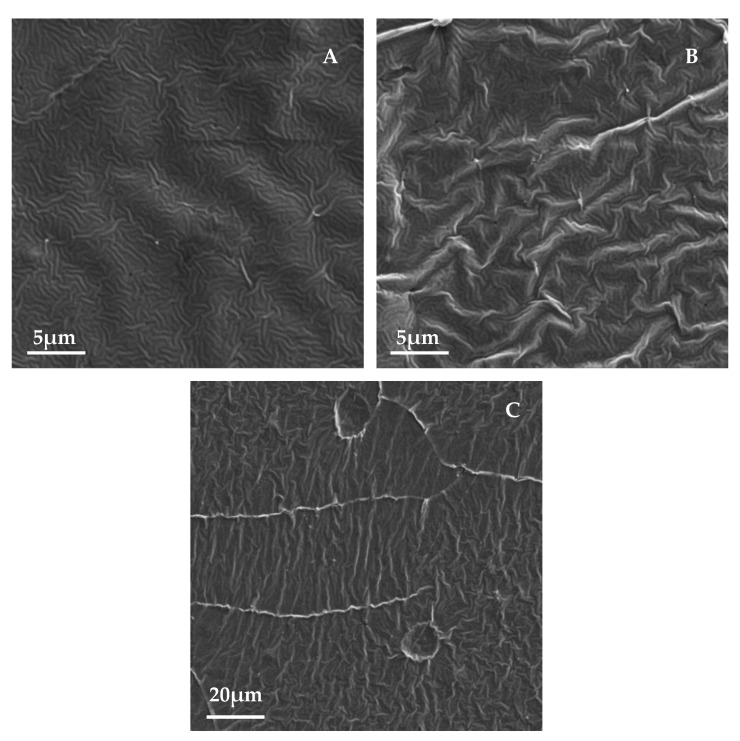
Image of the surface of the unheated sample taken by the SEM method. Layers of gold and titanium with a thickness of 10 and 20 nm, respectively, were deposited on the sample (**A**). The same sample after heating processing (**B**). SEM image of the heated sample with a gold bottom layer and a titanium top layer of 2 and 28 nm thicknesses, respectively (**C**).

Figure 2 shows the surfaces of the samples with the same combinations of Au and Ti thicknesses as in the previous part, but these samples were subjected to thermal stress at 60 °C for 1 h. The images show a formation of a wrinkled structure, similar to the ones in a previous study by Jurik et al. [7,8]. At the same time, the microcrack-like structure was partially preserved, which, however, can also take on the character of cross-linked bends (see Figure 3), which are visible on images from SEM—samples as bright raised lines. For this reason, in the following text, these structures will be discussed as “bends,” although in some images they may appear as simple “cracks” in the structure. The influence of these structures on the directionality of the waves is visible. In Figure 2a, in which this network is the densest, the ripples that formed were more disordered (Figure 2d). A similar ordered orientation of corrugations was visible even with certain combinations of metal thicknesses on samples with a titanium base layer. The stress-induced sputtering with high energy atoms in combination with slightly elevated temperature during the metallization process led to the formation of a primary randomly oriented wrinkled structure over a longer period of metal deposition. This structure is shown in Figure 3 (SEM analysis), which shows the surface of a sample with a 10 and 20 nm layer of gold and titanium, respectively.

Figure 3B shows the same sample after heating. From this image, it is apparent that the primary wrinkles remained even after the sample heating when a secondary wrinkled structure was formed (images from laser confocal microscopy). The PLLA film was probably heated due to the energy of the incident atoms, especially in the case of Ti. This caused local surface heating, where a process very similar to thermal stress took place, although only in a thin surface layer and with a slight increase in temperature towards Tg. The directionality of the stress in the polymer film structure, which leads to the formation of oriented wrinkle-like patterns [7,8], was not significantly disturbed, as the subsequent thermal stress led to further significant changes in surface morphology, as shown below. The presence of the primary ripples formed directly during deposition does not have a significant effect on the formation of secondary ripples during the subsequent heating process. However, the direction of the wave pattern created by the subsequent thermal stress was influenced by the presence of bends on the sample surface, in which the wrinkling stress was anisotropic. Therefore, in the area around the bend, the ripples were oriented perpendicularly to its edges, while outside this area, they were oriented randomly, see Figure 3C.

### 3.2. Sample Surface Morphology by AFM Analysis

**Figure 4:**
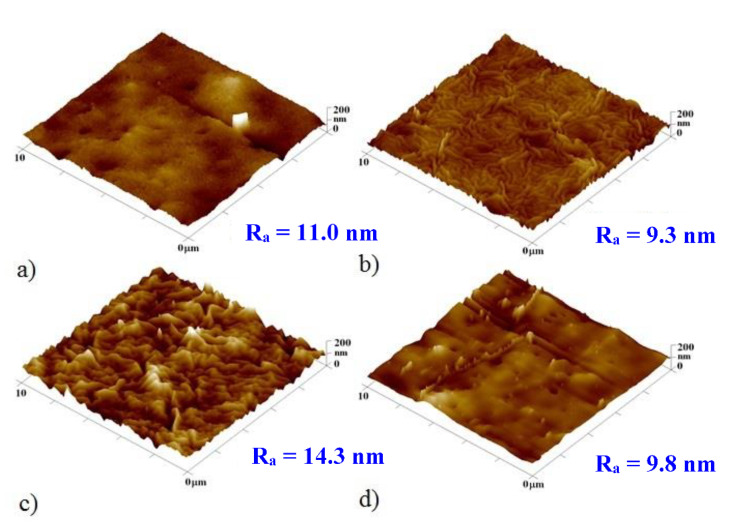
AFM images of the unheated PLLA samples with a sputtered titanium bottom layer and gold top layer. (**a**) Ti and Au layers of 2 and 48 nm, respectively; (**b**) Ti and Au layers of 5 and 45 nm, respectively; (**c**) Ti and Au layers of 10 and 40 nm, respectively; (**d**) Ti and Au layers of 25 and 25 nm, respectively. R_a_ is the mean surface roughness in nanometers.

**Figure 5:**
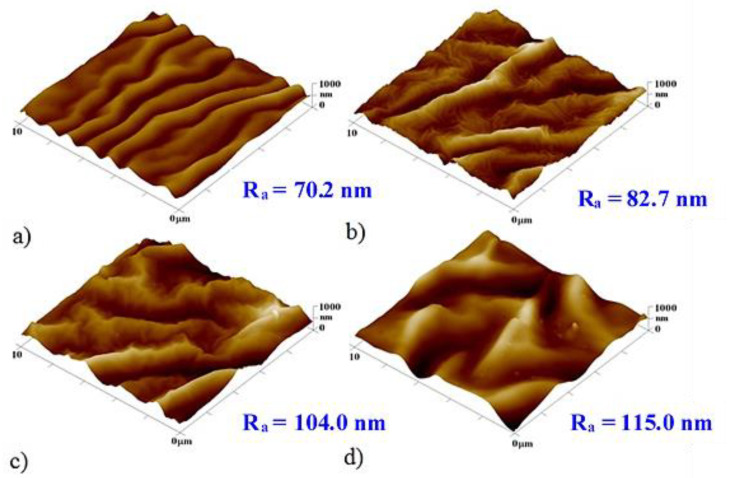
AFM images of the heated PLLA samples with a sputtered titanium bottom layer and a gold top layer. (**a**) Ti and Au layers of 2 and 48 nm, respectively; (**b**) Ti and Au layers of 5 and 45 nm, respectively; (**c**) Ti and Au layers of 10 and 40 nm, respectively; (**d**) Ti and Au layers of 25 and 25 nm, respectively. R_a_ is the mean surface roughness in nanometers.

Figure 4 and Figure 5 show AFM scans of selected unheated and heated PLLA samples with Ti and Au nanolayers together with a 2, 5, 10, and 25 nm thick titanium bottom layer. The PLLA surface with the lowest Ti thicknesses was evenly covered with a uniform metallic cluster array. The image with a 10-nm gold layer shows a disordered primary wrinkle structure accompanied by a slight increase in surface roughness. This slight increase indicates only a partial transformation in the process of wrinkle structures, the height of these waves being several tens of nanometers.

The samples with Au as the primary layer after heating have shown an intense increase in surface roughness with the formation of wavy structures caused by thermal stress. A significant effect of titanium on the surface roughness is evident. The PLLA sample with the highest thickness of a titanium layer (28 nm) also exhibited the largest surface roughness. As the proportion of the titanium layer decreased and the proportion of gold increased, so did the surface roughness. The sample with the biggest thickness of the gold layer (25 nm) and 5 nm layer of titanium had almost 50% of the roughness of the roughest sample. Sample a, with a 10-nm gold layer, retained the primary wrinkled structure formed by the deposition even after heating, which also increased the roughness of this structure. Figure 4 shows the surface of unheated samples with a titanium bottom layer and a gold top layer. The formed primary wavy structure is apparent in the images with 5 and 10 nm titanium layers. The corrugated surface with a titanium layer of 10-nm thickness exhibited the largest roughness of the whole sample series. Samples with a base titanium layer after heat treatment are shown in Figure 5. A wrinkled structure was formed by thermal stress induced by a temperature close to the Tg of the polymer. The sample roughness was also affected by the ratio of metals, increasing with a growing thickness of the titanium layer. On the sample with only 2 nm of titanium, the wrinkled structure exhibited a regular arrangement. Samples with a 5- and 10-nm titanium layer retained the primary ripples even after heating. The sample with a 25-nm titanium layer had the highest surface roughness compared to the others, but the ripples were not regular.

**Figure 6:**
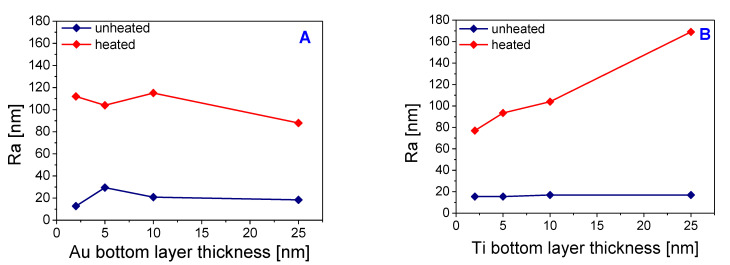
Surface roughness was determined by AFM on 30 × 30 μm images. Selected PLLA samples with sputtered Au (bottom layer) and Ti (top layer) layers with a total thickness of 30 nm (**A**). Surface roughness was determined by AFM on 30 × 30 μm images. Selected PLLA samples with sputtered Ti (bottom layer) and Au (top layer) layers with a total thickness of 50 nm (**B**).

Figure 6A,B represent the surface roughness values determined on the samples with different combinations of gold and titanium layer, while the sum of the deposited thickness remained constant and one of the deposited metals was changed. There is a significant roughness increase for heated samples due to the formation of the wrinkled pattern. For samples with a base gold layer, the surface roughness decreased with reducing the thickness of the upper titanium layer, which was especially evident for heated samples. The trend was the same for samples with a base layer of titanium. With its increasing thickness, there was a significant increase in surface roughness.

**Figure 7:**
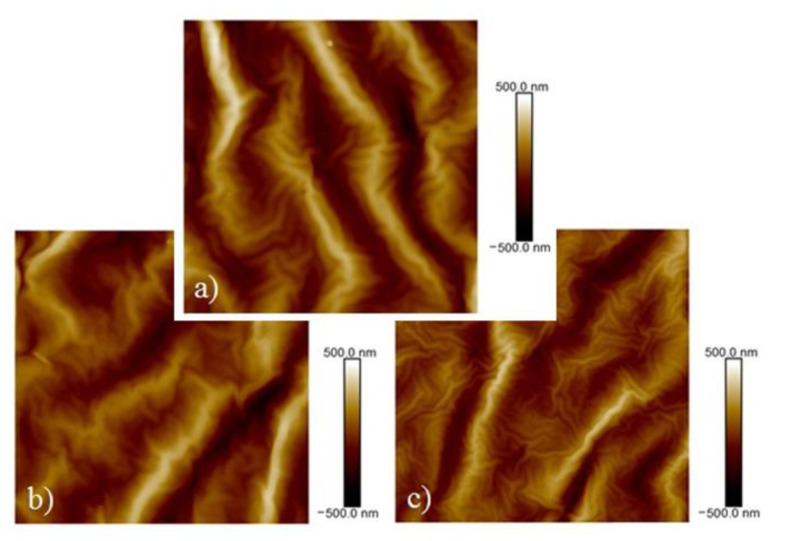
AFM images of heated samples with the size of 10 μm. (**a**) Ti and Au layers of 20 nm and 10 nm, respectively; (**b**) Ti and Au layers of 10 nm and 40 nm, respectively; (**c**) Ti and Au layers of 5 and 45 nm, respectively.

The surface morphology was different for each sample. While the sample with the bottom layer of gold of 10-nm thickness formed a wrinkled structure over the entire surface, the sample with the bottom layer of titanium of the same thickness created irregular wrinkling and also individual metal clusters were formed over the surface layer. The highest roughness values were obtained for a sample with a 10-nm thick gold bottom layer and a top layer of titanium of 20-nm thickness with subsequent heating. A comparison of the determined dimensions of the corrugations and the thicknesses of the deposited layers shows that the dimensions of the primary corrugated structure depend more on the type of a deposited metal than on the total applied thickness. As titanium thickness increased, the dimensions of the waves expanded as well. Figure 7 shows the morphology of the selected heated samples with different combinations and sequences of the metals. The morphology of the primary ripples induced by the sputtering process in combination with the secondary wrinkling structure formed after the heating process is also apparent. As also introduced in Figure 6, the surface roughness of the heated samples increases with the growing amount of titanium. All width and amplitude valus of the wrinkle are included in Table 2.

### 3.3. Surface Chemistry

**Figure 8:**
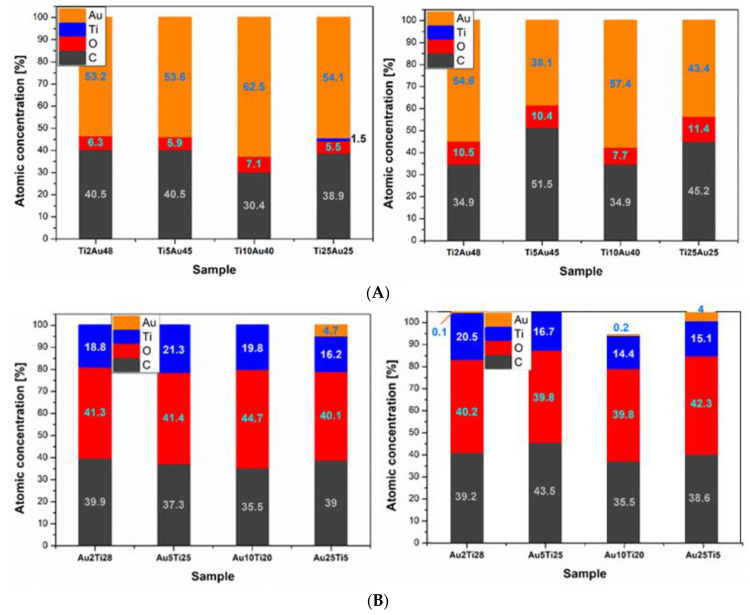
The concentration of the individual elements contained in the surface layer of the samples with the lower layer of titanium and the upper layer of gold obtained by XPS. (**A**) The results for samples with titanium bottom layer and gold upper layer before and after the heating process. (**B**) The results of the samples with the gold bottom layer and the titanium upper layer before and after the heating process. Unheated samples on the left, samples after heating on the right.

The chemical composition of the surface layer was determined by the XPS method. Figure 8A shows the results for samples with titanium bottom layer and gold upper layer before and after the heating process. Due to the relatively high thickness of the gold (25, 40, 45, and 48 nm), the presence of titanium appeared only on the samples with a Ti thickness of 25 nm, and the concentration of gold on the surface was about 55%. After heating, only a slight increase was detected in the concentration of carbon and oxygen from the polymer, probably due to morphological changes.

**Figure 9:**
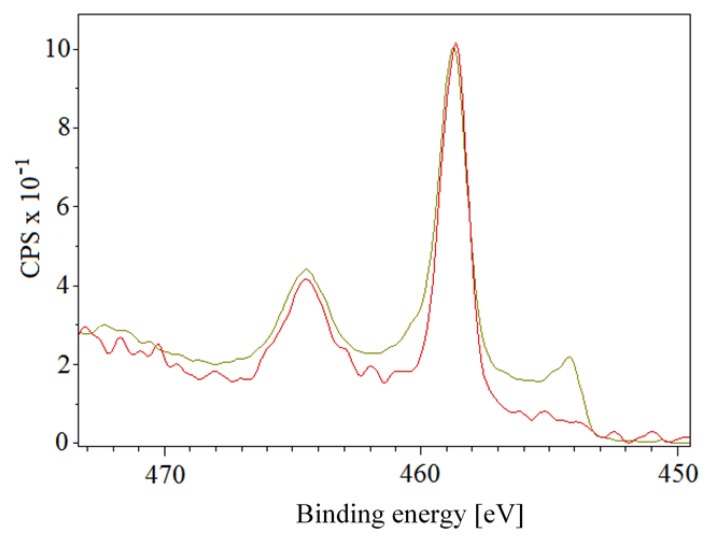
Representation of titanium oxide in the surface layer determined by XPS; two different angles, 9°, and 90°, were used for an unheated sample with sputtered 2 and 28 nm layers of gold and titanium, respectively. The red and green curves show measurements at the angles of 9° and 90°, respectively.

The results of the samples with the gold bottom layer and the titanium upper layer are shown in Figure 8B. The thickness of the upper titanium layer was 5, 20, 25, and 28 nm. In the case of unheated samples, the presence of gold appeared only for the samples with the greatest Au thickness of 25 nm. The concentration of titanium was about one-third compared to samples with a gold surface. At the same time, there was a much higher proportion of oxygen, which indicates the oxide layer formed in the Ti nanolayer. A slight decrease in titanium concentration was observed on heated samples. The presence of pure metal or titanium dioxide was examined in more detail. Figure 9 shows measurements at two angles (9° and 90°) for an unheated sample with a sputtered 2-nm gold layer and 28 nm of titanium. It is apparent that when measuring at a sharp angle (9°, red curve), only titanium dioxide with a binding energy of about 459 eV was detected, while at a perpendicular angle measurement, a metal with a binding energy of about 454 eV also appeared in the analyzed layer. The measurement, thus, shows that titanium metal can also be found at a very small depth below the surface layer of the oxide. In a similar comparison for the other samples with the top layer of titanium, the presence of metal in the surface layer was detected on both unheated and heated samples, except for the combination of 25 nm of gold and 5 nm of titanium, where only the titanium oxide layer was detected.

The composition of the sample in weight and atomic percentages was also monitored by EDS. Samples with a bottom layer of gold or titanium with a thickness of 5, 10, and 25 nm were measured, either as-sputtered or after the heating process. The results of the EDS analysis are shown in Table 3 and Table 4. For both series of samples, the increasing thickness of the lower metallic layer and the changing ratio of the deposited films led to a corresponding change in the percentage of gold and titanium. The heat treatment of samples had no significant effect on the atomic concentration of the metals, even though the surface morphology was changed drastically and the glass transition temperature of the polymer was reached. This phenomenon was probably due to the depth of reach of this analytical method, which extended up to 50 nm, in contrast to the XPS method, where it was about 1 nm and, therefore, even changes on the very surface were determined by XPS; no such dramatic changes were determined by EDS.

### 3.4. Sample Wettability and Its Zeta-Potential

**Figure 10:**
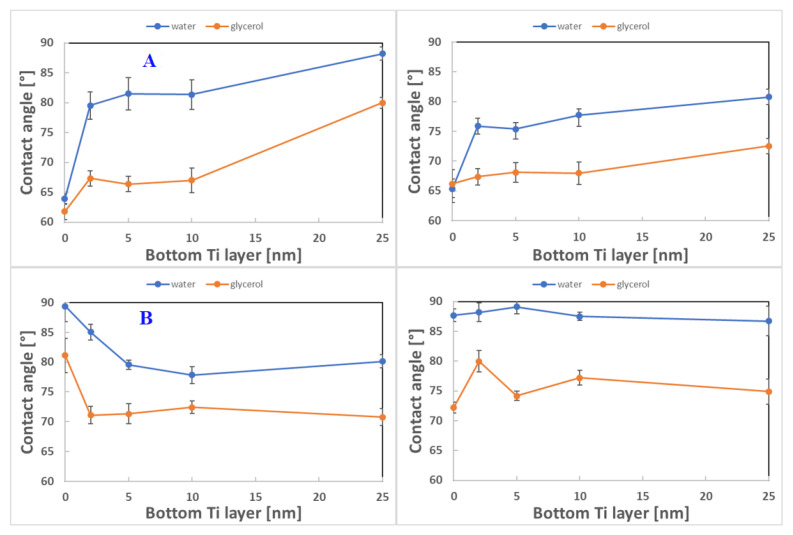
(**A**) The contact angle of the modified samples with the lower layer of titanium and the upper layer of gold with a total thickness of 50 nm. Unheated samples on the left, heated samples on the right. (**B**) The contact angle of modified samples with a bottom layer of gold and a top layer of titanium with a total thickness of 30 nm. Unheated samples on the left, heated samples on the right.

Figure 10A shows the change in wettability for unheated and heated samples with a titanium bottom layer and a gold upper layer with a total thickness of 50 nm. For a sample, at which 50 nm of gold was deposited without a titanium bottom layer, the measured water contact angle was 63.8°; for glycerol it was 61.8°. The influence of the lower layer of titanium was already very significant when its thickness reached 2 nm. For samples with 2 nm of titanium as the upper layer and unheated samples, the water contact angle increased by 25%; a less significant increase was observed for glycerol. The increasing thickness of the titanium layer led to an increase in the contact angle for both liquids. The presence of a titanium bottom layer in the samples induced a decrease in the water contact angle for all studied samples. The opposite effect was observed for samples with a gold bottom layer (Figure 10B). For subsequently deposited Ti, the water contact angle decreased for a lower Ti layer, followed by contact angle restoration at values still lower than for deposited samples without Ti. The subsequent heating of PLLA with an Au bottom layer stabilized the water contact angle at a narrow interval of 86–90°.

**Figure 11:**
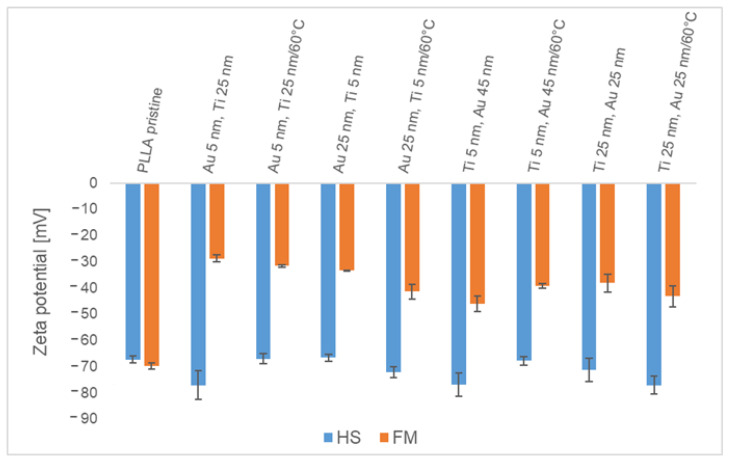
Zeta potential of sputtered samples with Au and Ti layers of different thicknesses and the same heated samples determined using the Helmholtz–Smoluchowski (HS) and Fairbrother–Mastins (FM) equations.

The results of the zeta potential measurements are shown in Figure 11. The values were obtained for gold and titanium bottom layer thicknesses of 5 and 25 nm, respectively, both for unheated and heated samples. The unheated samples with 5-nm gold and 25-nm titanium layers and with 5-nm titanium and 45-nm gold layers had a lower zeta potential compared to unmodified PLLA. In both cases, an increase in the thickness of the metallic bottom layers led to an increase in the zeta potential. The heated samples with a 5-nm metallic bottom layer had a higher zeta potential compared to unheated samples; the increase in thickness of the bottom layer led to a decrease in the zeta potential. For unmodified PLLA, the values did not vary significantly. If we compare the determined zeta potential and contact angle, it can be concluded that with an increasing contact angle, the zeta potential increased for unheated samples with a gold bottom layer as well as with samples with a titanium bottom layer.

### 3.5. Antibacterial Activity of the Samples

**Figure 12:**
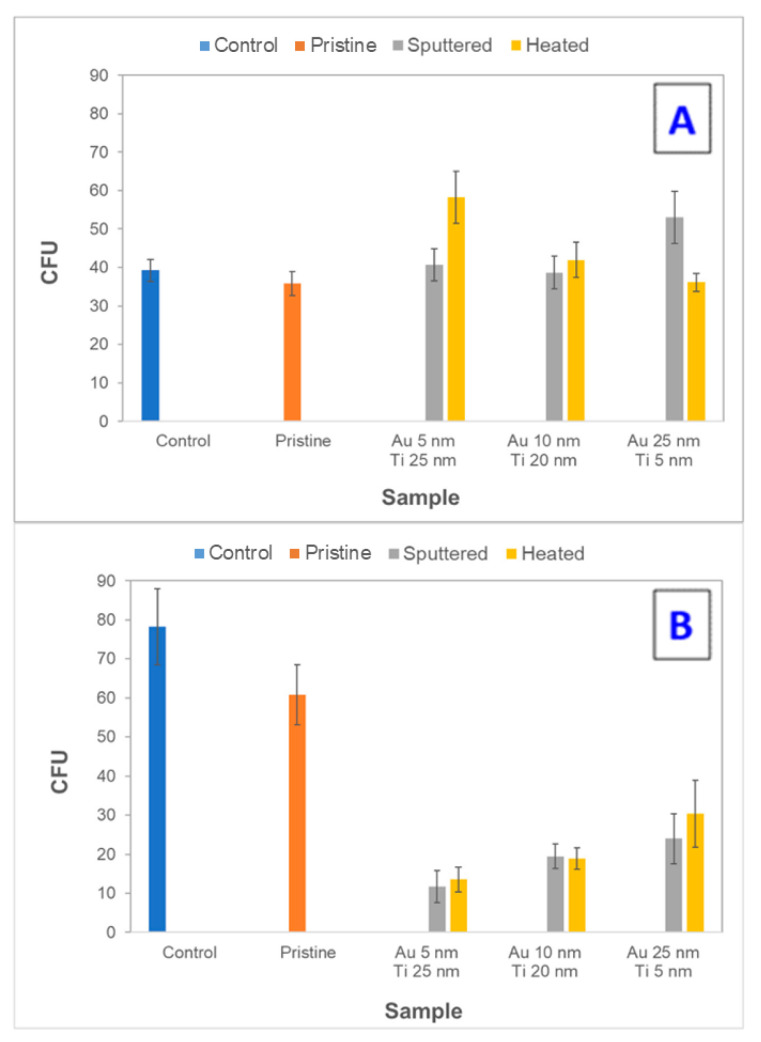
The number of colony-forming units (CFU) of Gram-negative bacterial strain of *E. coli* incubated with the samples with Ti and Au layers of different thicknesses. (**A**) 2 h, and (**B**) 24 h of contact of the bacterial suspension with the evaluated samples. The blue column represents the CFU of control (untreated bacteria), the orange column represents the CFU of bacteria incubated with a pristine sample, the grey, and yellow columns represent the CFU of bacteria incubated with Ti and Au layers (various thickness), respectively.

Antibacterial properties were monitored on unheated and heated samples with a top Ti layer with thicknesses of 5, 20, and 25 nm. For comparison, the CFU of *E. coli* was determined for unmodified PLLA (designated pristine) and in the control group. A decrease in the CFU of *E. coli* was evaluated after 2 and 24 h of incubation with the samples. These two incubation periods were selected to determine the immediate and long-term antibacterial activity. The results are shown in Figure 12A,B. After a 2-h incubation of the samples with *E. coli*, it was apparent that there was no immediate antibacterial effect. However, the *E. coli* colonies grown on agar plates after incubation with samples of 20- and 25-nm thick titanium layers in the surface were about half the size of the untreated control (*E. coli* in physiological solution for 2 h), even though the number of CFU was comparable for the samples as well as the controls. The smaller colonies suggest that there was a bacteriostatic effect. The heating of the samples did not affect the antibacterial activity in any manner.

However, the samples showed a significant antibacterial effect after 24-h incubation (in contact) with the *E. coli* bacterial suspension. The results of the measurements are shown in Figure 12B. The partial antibacterial activity was also manifested on unmodified PLLA. However, the effect was much more pronounced on modified polymer samples. With the increasing thickness of the upper titanium layer, a pronounced antibacterial effect was detected. Additionally, it can be concluded that for samples with “thicker” Ti layers, there was no noticeable effect of heating. The observed decrease in the number of CFU of *E. coli* during 24-h exposure of the samples compared to the control group is summarized in Table 5. The antibacterial effect is supported by the presence of TiO_2_ (Figure 9), where despite that, no further excimer irradiation (ultraviolet wavelength) was performed; the ultraviolet part of the ambient irradiation likely supported the results.

**Figure 13:**
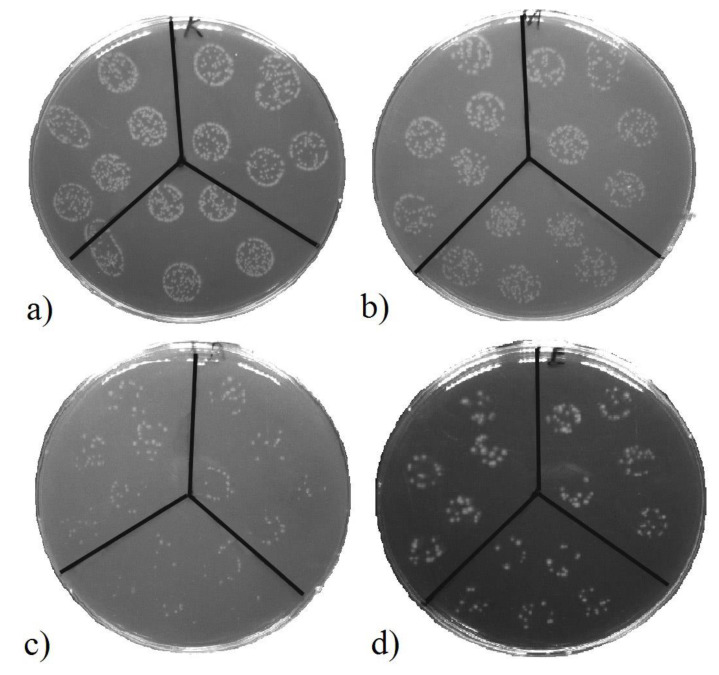
Growth reduction of *E. coli* on evaluated samples after 2 h of incubation. Images of agar plates with *E. coli* colony-forming units. (**a**) Control (incubated only in physiological solution); (**b**) Pristine (PLLA); (**c**) Unheated sample with 5 and 25 nm of Au and Ti layers, respectively; (**d**) Heated sample with 5 and 25 nm of Au and Ti layers.

Figure 13 represents images of *E. coli* CFU on LB agar plates inoculated after incubation with selected samples. Figure 13 shows a reduction in bacterial growth after 24 h of contact with PLLA-modified samples. The sample, which was thermally stressed, exhibited an effect on the shape and size of the bacterial colonies. Compared to other images, the boundaries of the colonies are blurred. The same difference in shape for the bacteria that were in contact with the samples was observed for all combinations of metal thicknesses used before and after heating. The photocatalytically active layer of titanium dioxide formed on their surface after sputtering due to contact with air, which enhanced the antibacterial properties of the samples. Because daylight with no further irradiation supplies the samples with relatively low energy and TiO_2_ is present in the sample in small amounts, the photocatalytic activity was not very high. After 2 h of contact, no bactericidal effects were observed and, therefore, a longer, 24-h treatment was required. The results, thus, agree with the data found in the literature.

## 4. Conclusions

Heat treatment of the PLLA with thin metallic layers (Au/Ti, Ti/Au) to the polymer Tg led to the formation of a wrinkled pattern, which was caused by relaxation of the stress present inside the polymer film during heating. The structure showed an ordered direction over larger areas. The stress release in the polymer occurred already during sputtering, probably due to the high energy of titanium. This relaxation was manifested by various mechanisms, namely the formation of secondary structures, for example, cross-linked bends, structures with higher porosity compared to other samples, or randomly oriented primary wavy structures with low surface roughness. The formation of a wrinkled structure after heat treatment of the polymer led to an intense increase in the surface roughness of all samples. The roughness was affected by the type of deposited metal, where titanium leads to significantly higher values of surface roughness than the gold of the same thickness. XPS analysis of the surface layer composition showed the formation of a titanium dioxide layer on samples with a deposited gold bottom layer and titanium upper layer. The titanium layer deposited below the gold layer led to a decrease in wettability, while the contact angle for water and glycerol increased with the growing thickness of a titanium layer. After longer action of 24-h incubation with the samples, the decrease in CFU of *E. coli* was up to 85% (compared to control) for PLLA with a titanium layer of the biggest thickness. Overall, the antibacterial effect significantly increased with the growing amount of Ti on the surface. The bactericidal properties were induced by the formation of titanium dioxide on the surface of the modified PLLA, which was confirmed by XPS analysis. As a future outlook, we plan to combine titanium with other noble metals, such as silver and palladium, or titanium and carbon nanolayers. Moreover, we plan to study the wrinkling phenomenon for these combinations as well as the antibacterial properties of activated surfaces. Additionally, we plan to study the influence of a high-energy excimer laser on the prepared pattern.

## Figures and Tables

**Table 1:** Overview of the prepared samples. Two series with the opposite order of deposited metals (titanium, gold) were prepared. In each series, four types of samples differing in the ratio of the thicknesses of the metallic layers were prepared.

Ti Base Layer		Au Base Layer	
Ti [nm]	Au [nm]	Au [nm]	Ti [nm]
2	48	2	28
5	45	5	25
10	40	10	20
25	25	25	5

**Table 2:** The average values of amplitudes and widths of wrinkles generated by sample sputtering; determined from 3 × 3 μm AFM images.

Sample	Amplitude [nm]	Width [μm]
Ti 5 nm, Au 45 nm	18.7 ± 2.8	0.25 ± 0.03
Ti 10 nm, Au 40 nm	27.4 ± 4.1	0.32 ± 0.06
Au 10 nm, Ti 20 nm	59.8 ± 9.5	0.45 ± 0.03

**Table 3:** The concentration of individual elements contained in unheated and heated samples with a lower layer of gold and a top layer of titanium obtained by EDS. Values for mass (Wt) and atomic (At) percentages.

Sample	C		O		Ti		Au	
**Unheated**	**Wt %**	**At %**	**Wt %**	**At %**	**Wt %**	**At %**	**Wt %**	**At %**
Au 5 nm, Ti 25 nm	82.5	90.5	9.4	7.8	6.0	1.7	2.0	0.1
Au 10 nm, Ti 20 nm	72.5	80.3	22.7	18.9	2.3	0.7	2.5	0.2
Au 25 nm, Ti 5 nm	70.6	83.9	16.6	14.8	1.6	0.5	11.2	0.8
**Heated**	**Wt %**	**At %**	**Wt %**	**At %**	**Wt %**	**At %**	**Wt %**	**At %**
Au 5 nm, Ti 25 nm	81.9	89.8	10.4	8.5	5.9	1.6	1.8	0.1
Au 10 nm, Ti 20 nm	73.2	81.2	21.5	17.9	2.4	0.7	2.8	0.2
Au 25 nm, Ti 5 nm	69.5	83.1	17.7	15.6	1.6	0.5	11.2	0.8

**Table 4:** The concentration of individual elements contained in unheated and heated samples with a lower layer of titanium and a top layer of gold obtained by EDS. Values for mass (Wt) and atomic (At) percentages.

Sample	C		O		Ti		Au	
**Unheated**	**Wt %**	**At %**	**Wt %**	**At %**	**Wt %**	**At %**	**Wt %**	**At %**
Au 5 nm, Ti 25 nm	62.5	88.3	8.4	8.9	0.9	0.3	28.1	2.4
Au 10 nm, Ti 20 nm	77.3	91.4	8.3	7.3	1.2	0.4	13.3	0.9
Au 25 nm, Ti 5 nm	71.5	87.4	11.1	10.2	4.9	1.5	12.5	0.9
**Heated**	**Wt %**	**At %**	**Wt %**	**At %**	**Wt %**	**At %**	**Wt %**	**At %**
Au 5 nm, Ti 25 nm	69.0	83.9	16.2	14.8	0.6	0.2	14.2	1.0
Au 10 nm, Ti 20 nm	67.1	89.3	8.3	8.3	1.9	0.7	22.7	1.8
Au 25 nm, Ti 5 nm	79.5	89.8	10.3	8.7	3.5	1.0	6.7	0.5

**Table 5:** Growth reduction of *E. coli* after 24 h of exposure to the evaluated samples in comparison to untreated control.

As-Sputtered Samples	Growth Reduction [%]	Heated Samples	Growth Reduction [%]
Pristine	22.3	-	-
Au 5 nm, Ti 25 nm	85.0	Au 5 nm, Ti 25 nm	82.7
Au 10 nm, Ti 20 nm	75.1	Au 10 nm, Ti 20 nm	75.8
Au 25 nm, Ti 5 nm	69.3	Au 25 nm, Ti 5 nm	61.2

## Data Availability

Not applicable.
